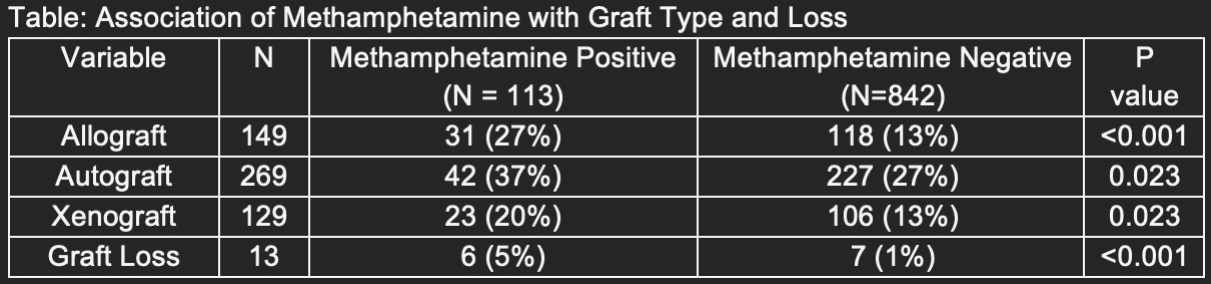# 519 A Burning Issue: Methamphetamine Intoxication Association with Graft Loss

**DOI:** 10.1093/jbcr/iraf019.148

**Published:** 2025-04-01

**Authors:** Parisa Oviedo, John Austin, Laura Haines, Jeanne lee, Jarrett Santorelli

**Affiliations:** University of California, San Diego Health Regional Burn Center; University of California, San Diego Health Regional Burn Center; University of California, San Diego Health Regional Burn Center; University of California, San Diego Health Regional Burn Center; University of California, San Diego Health Regional Burn Center

## Abstract

**Introduction:**

Methamphetamine intoxication can lead to serious medical complications in burn patients, including longer ICU stays, increased risk of DVT, sepsis and more burn debridement procedures. However, its impact on graft durability remains unexplored. We hypothesize that methamphetamine-positive burn patients have higher rates of graft use and graft loss.

**Methods:**

A single center retrospective cohort study from the years 2021-2024 was performed at an ABA verified burn center. The primary outcome was graft loss requiring return to operating room for re-graft or flap repair. Patient demographics, toxicology results, burn characteristics, and graft types were recorded, along with hospital, ICU, and ventilator days. Chi-square tests were performed for categorical variables, while Mann-Whitney U tests were performed for continuous variables.

**Results:**

955 patients were included in this study with median age 39 years and median total TBSA 4%. 113 patients tested positive for methamphetamine. Meth positive patients had higher rates of graft loss. They also had increased use of allograft, autograft, and xenograft. Meth positive patients presented primarily with flame burns (28.4%) while negative patients presented primarily with scald burns (42.5%). They also had increased age, increased total TBSA, longer hospital LOS, more days in the ICU, and mechanical ventilation (p< 0.001). On regression analysis, meth status was determined to be an independent risk factor for graft loss.

**Conclusions:**

Meth positive burn patients were more likely to sustain flame burns, undergo autograft or allograft or xenograft, and more likely to experience graft loss as a complication. Our study identifies meth use as a unique risk factor for burn patients and demonstrate they are at higher risk of failing graft management. Routine toxicology testing can promptly identify these patients and notify providers earlier to plan for more intensive burn care management.

**Applicability of Research to Practice:**

Use of grafts and skin substitutes is crucial to management of sick burn patients. This study identifies a particularly vulnerable patient population for developing graft complications. This population can be readily identified on routine admission toxicology testing. To guide burn education and prevention efforts, future studies should perform a geospatial analysis of burn characteristics on admission and explore socioeconomic disparities in meth positive burn patients.

**Funding for the Study:**

N/A